# Association between number of remaining teeth and incident depression in a rural Chilean cohort

**DOI:** 10.1186/s12903-023-03374-4

**Published:** 2023-09-04

**Authors:** Duniel Ortuño, Constanza Martínez, Constanza Caneo

**Affiliations:** 1 Faculty of Medicine, PhD in Epidemiology Program, Pontificia Universidad Católica de Chile, Santiago, Chile; 2 Facultad de Odontologia, Universidad de los Andes, Santiago de Chile, Chile; 3 School of Dentistry, Faculty of Medicine, Pontificia Universidad Catolica de Chile, Santiago de Chile, Chile

**Keywords:** Cohort, MAUCO, Number of teeth, Oral health, Epidemiology, Depression

## Abstract

**Objectives:**

Previous studies have established an association between tooth loss and depression. However, longitudinal evidence is scarce and needs to be verified in other populations. The aim of this study was to examine the longitudinal association between the number of remaining teeth and incident depression at 2- and 4-years follow-up in individuals enrolled in the Maule cohort (MAUCO) in Chile.

**Methods:**

This prospective study used the information of individuals, aged 38 to 74 years, excluding those with depression at baseline. The number of remaining teeth at baseline was determined in four groups: “20 or more teeth”, “10 to 19 teeth”, “1 to 9 teeth” and “no natural teeth”. Depression was measured through the PHQ-9. Logistic regression was performed to calculate the odds ratios (OR) for incidence depression at both periods of follow-ups, adjusting for age, sex, educational attainment, diabetes mellitus II, and stressful events at follow-up. Also, we performed adjusted multinomial logistic models to analysis the association between the number of remaining teeth and depression severity.

**Results:**

In total individuals (n = 3335 at follow 1, n = 2461 at follow 2), all groups have ORs for incident depression above 1 considering 20 or more teeth as reference. In men, those with 10–19 teeth have 2.44 times higher odds of incident depression than those with 20 or more teeth (OR 2.44, CI 95% 1.33–4.50). Edentulous subjects at 4 years follow-up had 2.24 times higher odds of depression than those with more than 20 teeth (OR 2.24 CI 95%1.35–3.72). In women, the ORs (CI 95%) of incident depression were 2.56 (1.50–4.39), 1.56 (1.02–2.40) and 1.27 (0.90–1.81) for “none”, “1–9”, “10–19” respectively in comparison to the reference group. In edentulous individuals at baseline, the odds for each of the comparisons “mild vs no”, “moderate vs no”, “moderately severe vs no” and “severe vs no” were above 1, at both follow-ups.

**Conclusion:**

Individuals with less than 20 teeth in the mouth could had higher odds of incident depression at 2- and 4-years follow-up, with differences between men and women. Also, in our study, edentulism was associated with increased odds of incident depression at 4-years follow-up in women, and with higher levels of severity of depressive symptoms.

**Supplementary Information:**

The online version contains supplementary material available at 10.1186/s12903-023-03374-4.

## Background

Depression is a common illness that severely limits psychosocial functioning and diminishes the quality of life [1]. It occurs at any stage of the life cycle and tends to manifest itself with the appearance of several episodes during life. The onset of depression is usually gradual, and the duration of depressive symptoms varies considerably [1]. The symptoms of depression can be grouped into neurovegetative, emotional, and cognitive, and the detection can be challenging [1]. Screening tools have been developed to identify depression in various clinical settings, and some self-report instruments can be used in a waiting room or online [2]. The World Health Organization ranked depression as fifth among diseases with the highest burden, especially in developed countries [3]. The 12-month prevalence of major depressive disorder varies considerably across countries but is approximately 6% overall [4]. Also, due to COVID-19, there was an estimated globally increase in depression cases of 28% (95% CI = 27.2–28.9) [5].

Oral diseases are another major public health issue affecting more than 3.5 billion people worldwide [6]. According to cross-sectional studies, bidirectional associations between oral health and depression have been suggested. The relationship between depression and oral health could be explained by lifestyle changes, poor oral hygiene, and difficulties accessing dental care [7–9]. A systematic review showed that the history of caries lesions was higher in individuals with depression, who had an odds of 2.8 (CI 95%=1.7–4.6) of having lost all their teeth than the general community [8]. Okoro et al. (2012) found that the adjusted odds of being in the “1–5 teeth removed” or “6–31 teeth removed” groups compared to “0 teeth removed” was also increased for adults with lifetime diagnosed depression versus those without this disorder [10]. Also, these authors reported that adults with current depression had a higher prevalence of nonuse of oral health services in the past year, considering age, sex, ethnicity, education, and chronic conditions like diabetes mellitus II [10]. A cohort study concluded that the risk of periodontitis was 19% greater in individuals with depressive symptoms (RR 1.19; CI 95%=1.04–1.36), independent of oral hygiene and systemic inflammation [11]. However, other studies did not find an association between depression and periodontitis [12].

The presence of oral conditions such as tooth loss affects general health, including mental health status and health-related quality of life [13]. One study showed that individuals with six or more teeth removed were at higher risk of depression alone (OR 1.64, CI 95%= 1.52–1.77) or with anxiety comorbidity (OR 1.91, CI 95%= 1.72–2.11) [14]. Tyrovolas et al. (2016) concluded that edentulism was significantly associated with depression (OR 1.57, CI 95%= 1.23-2.00) in the younger group with no significant associations in the older age group [15]. Additionally, the consequences of missing teeth depend on the severity and intraoral location [16]. A recent study concluded that tooth loss causally increased depression among US adults, but the findings may not be directly transferrable to other countries or settings [17]. The aim of this study was to examine the longitudinal association between the number of remaining teeth with incident depression at 2- and 4-years follow-up in individuals enrolled in the Maule cohort (MAUCO) in Chile. We hypothesized an association between a fewer number of remaining teeth with increased risk of incident depression in adults at both follow-ups.

## Methods

### Study population

This longitudinal prospective study used the information of individuals, aged 38 to 74 years (53.5, sd 9.8 at baseline), enrolled in the Maule cohort (MAUCO) during 2014–2017. MAUCO is the first prospective population-based cohort of cardiovascular disease (CVD) and cancer in central Chile. The study protocol for the MAUCO was previously published [18]. To be included in the cohort, participants had to meet the residency criteria and give informed consent. Individuals must have resided in Molina for at least 6 months prior to enrolment and not have plans to move from this town in the next 3 years. Molina is a small agricultural county with 42,859 inhabitants, 30.1% living in rural areas. Individuals who could not give informed consent autonomously or who has a terminal illness were excluded. Also, individuals without the completion of the Patient Health Questionnaire depression module (PHQ-9) or completed dental examination upon entry into the cohort were excluded. Accepting participants received the first interview about lifetime health information and physical examination in their homes or at the MAUCO study clinic. Contact was established in 70% of visited households, and in 95% of those, a resident agreed to respond to the survey. In MAUCO, follow-up visits were planned at 2,4 and 8 years after enrolment [18].

### Outcome variable

Depression was measured at baseline and follow-ups in all subjects through the PHQ-9, a nine-item questionnaire, each rated from 0 to 3 on a Likert scale, where 0 is “not all present” and 3 is “nearly every day”, resulting in a total score that ranges from 0 to 27 [19, 20]. It is designed to measure severity of symptoms, where higher score means increased severity and a greater risk of major depressive disorder. The PHQ-9 was validated by Baader et al. (2012) in Chile by assessing 1327 patients in urban primary care practices in Valdivia County in southern Chile [21]. Those investigators used a cut-off score of 10 or higher on the PHQ-9 as positive for the presence of depression, which is the cut-off score used in our study, so individuals with baseline depression (PHQ-9 ≥ 10) were excluded. Additionally, we considered the severity of depression as outcome according to the following PHQ-9 score levels: no (scores of 0–4), mild (scores of 5–9), moderate (scores of 10–14), moderately severe (scores of 15–19) and severe depression (scores of 20–27) [19]. PHQ-9 was applied by trained health technicians either at the participant’s home or at the MAUCO study clinic. Data entry, supervised by the data-manager, was done locally using REDCap™ (Research Electronic Data Capture) [18].

### Independent variable

The number of remaining teeth at baseline was counted and categorized into the following four groups: “20 or more teeth”, “10 to 19 teeth”, “1 to 9 teeth” and “no natural teeth”. Trained health technicians, using basic dental instruments and after asking the patient to remove their prosthesis, determined the number of remaining teeth, which was registered in an odontogram. Dental status was assessed using the standard diagnostic criteria of the World Health Organization [22]. The health technicians wore gloves, a surgical mask, a head flashlight, tongue depressors, and flat mouth mirrors.

### Confounders

**Fig. 1:**
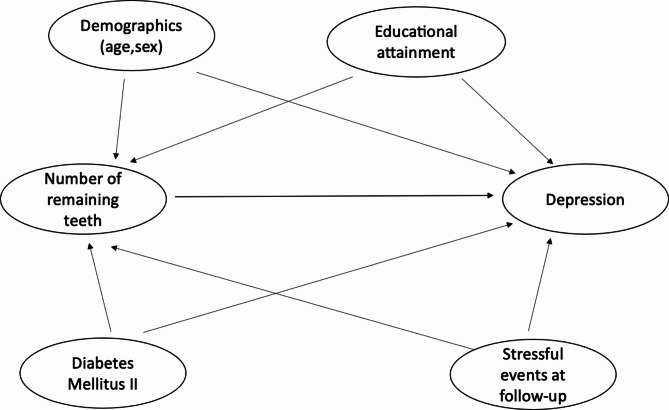
The hypothesized association between number of remaining teeth and depression, including the confounders. Demographics included sex and age. Stressful events at follow up included one of these conditions after baseline: 1-divorce or separation, 2- job loss or retirement. 3- business failure, 4- violent event 5- major family problem, 6- recent health problem or accident, 7- death of spouse or partner, 8- illness or death of close relative, and 9- other major stress situation. *DAGitty version 3.0*

A directed acyclic graph (DAGitty version 3.0) was performed to structure the theorical framework of this study (Fig. 1). Following a conservative approach for selecting the confounders variables, the data collected at the baseline for demographics, educational attainment and diabetes mellitus II was considered in our DAG. Sex and age were considered as demographic confounders. Educational level was determined by years of formal education: ≥12 years of formal Education, 9–11 years of formal Education ≤ 8 years of formal education. Also, diabetes mellitus II was defined by self-report or glycaemia ≥ 126 mg/dL or use of hypoglycemic drugs. Another variable included was stressful events at follow-up, which was the result of the presence (yes/no) of at least one of these conditions after baseline: 1-divorce or separation, 2- job loss or retirement. 3- business failure, 4- violent event 5- major family problem, 6- recent health problem or accident, 7- death of spouse or partner, 8- illness or death of close relative, and 9- other major stress situation.

### Statistical analysis

A descriptive analysis was used examine the baseline number of remaining teeth and the descriptive statistics of depression severity of individuals at 2- and 4-years follow-up. Logistic regression was performed to calculate the odds ratios (OR) and respective confidence intervals (95%) for incidence depression at both periods of follow-ups. In this analysis depression was a binary outcome, being yes, a PHQ-9 ≥ 10 and not a PHQ-9 < 9. Logistic regressions were performed to obtain ORs of incident depression in women or men. A second approach was used to analysis depression severity utilizing multinomial logistic regressions. In all models, individuals with baseline depression (PHQ-9 ≥ 10) were excluded. We only showed fully adjusted models. Statistical Analyses were performed using Stata/SE 16 software from StataCorp LP, and the STROBE guidelines for cohort studies were followed by the authors.

### Ethical approval

The MAUCO protocol was approved by the ethics committees of Pontificia Universidad Católica de Chile and the Maule Regional Service of the Chilean Ministry of Health. Participants were notified that participation was voluntary. Informed consent was obtained from all subjects. The participants’ privacy and data were protected throughout the study and individuals with abnormal results were informed and referred to their health care system accordingly [18].

## Results

**Fig. 2:**
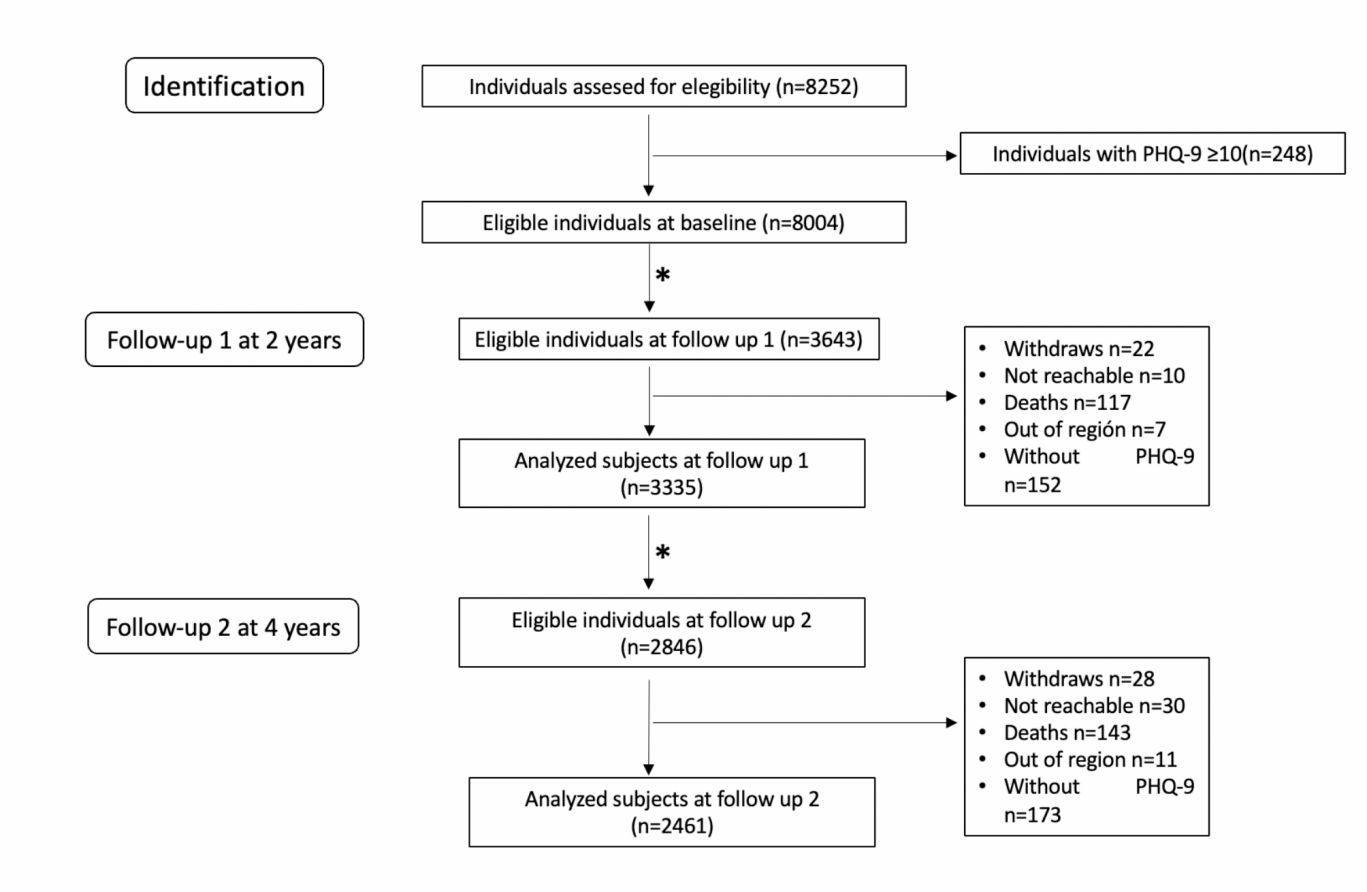
The participants flowchart of analytic sample (n _*follow−up 1*_ = 3335)(n _*follow−up 2*_ = 2461) *a subgroup of the cohort underwent complete examinations like baseline at follow up

Figure 2 shows the flow chart of the participant´s inclusion. Finally, 3 335 individuals were included at follow-up 1 (two years) and 2 461 at follow-up 2 (four years). Table 1 showed the descriptive statistics of the participants depression severity at 2- and 4-years follow-up, according to the number of remaining teeth at baseline. Those with higher number of teeth (10–19, ≥ 20) at baseline tended to be less depressive at follow up, while those with no teeth were more severely depressed.

**Table 1:** Descriptive statistics of the number of remaining teeth at baseline and depression severity at follow up 1 (2 years) and follow up 2 (4 years), excluding individuals with depression at baseline (PHQ-9 > 10).

number of remaining teeth (*baseline)*	*follow-up 1 (2 years) Depression severity*					total
	no (n/ %)	Mild (n/ %)	Moderate (n/ %)	moderately severe (n/ %)	severe (n/ %)	
none	154/ 58.8	67/ 25.6	22/ 8.4	12/ 4.6	7/ 2.6	262
1–9	375/ 67.5	99/ 17.8	42/ 7.5	30/ 5.4	10/ 1.8	556
10–19	529/ 61.2	195/ 22.5	95/ 11.0	28/ 3,2	18/ 2.1	865
≥ 20	1 086/ 65.7	345/ 20.9	141/ 8.5	57/ 3.5	23 /1.4	1 652
total	2 144/ 64.3	706/ 21.2	300/ 9.0	127/ 3.8	58/ 1.7	3 335
	*follow-up 2 (4 years) Depression severity*					
none	118/ 62.5	38/ 20.1	18/ 9.5	11/ 5.8	4/ 2.1	189
1–9	299/ 68.6	82/ 18.8	37/ 8.5	14/ 3.2	4/ 0.9	436
10–19	469/ 69.3	130/ 19.2	47/ 6.9	25/ 3.7	6/ 0.9	677
≥ 20	775/ 66.9	253/ 21.8	84/ 7.3	34/ 2.9	13/ 1.1	1 159
total	1 661/ 67.5	503/ 20.4	186/ 7.6	84/ 3.4	27/ 1.1	2 461

Table 2 contained the findings of the logistic regression analysis of the number of remaining teeth at the baseline and the incident depression at 2- and 4-years follow-up of our cohort. For the first follow-up period for the total and both sexes, we observed higher odds of incident depression in the group with 10–19 teeth compared to more than 20 teeth. When considering total individuals, all groups have ORs for incident depression above 1 when considering 20 or more teeth at baseline, but these differences were not statistically significant for most groups. In the case of men, those with 10–19 teeth have 2.44 times higher odds of incident depression than those with 20 or more teeth at baseline (OR 2.44, CI 95% 1.33–4.50). In relation to 4-years follow-up, those who had fewer teeth tended to have higher odds of being depressed compared to the reference group (≥ 20 teeth at baseline), considering comparisons for total and women. Edentulous subjects (“none” teeth) at 4 years follow-up had 2.24 times higher odds of depression than those with more than 20 teeth in the mouth at baseline (OR 2.24 CI 95%1.35–3.72). In women, the ORs (CI 95%) of incident depression were 2.56 (1.50–4.39), 1.56 (1.02–2.40) and 1.27 (0.90–1.81) for “none”, “1–9”, “10–19” respectively in compared to the reference group (≥ 20 teeth at baseline).

**Table 2:** Summary of findings of the logistic regression analysis of the number of remaining teeth at the baseline and the incident depression follow-up 1 (2 years) and follow-up 2 (4 years)

number of remaining teeth *(baseline)*	*follow-up 1 (2 years) Incident Depression*		
	Total (OR and CI 95%)	Women (OR and CI 95%)	Men (OR and CI 95%)
none	1.06 (0.70–1.62)	0.93 (0.59–1.47)	2.16 (0.69–6.74)
1–9	1.13 (0.82–1.56)	1.01 (0.71–1.44)	2.07 (0.91–4.72)
10–19	1.29 (1.00-1.66)	1.12 (0.84–1.48)	2.44 (1.33–4.50)
≥ 20	1	1	1
	*follow-up 2 (4 years) Incident Depression*		
none	2.24 (1.35–3.72)	2.56 (1.50–4.39)	0.79 (0.15–4.13)
1–9	1.45 (0.97–2.17)	1.56 (1.02–2.40)	0.87 (0.29–2.67)
10–19	1.16 (0.84–1.61)	1.27 (0.90–1.81)	0.62 (0.25–1.54)
≥ 20	1	1	1

The model was adjusted for age, sex, educational attainment, diabetes mellitus II and stressful event at follow-up. OR: odds ratio. CI: confidence interval

Table 3 showed the findings of the multinomial logistic regression analysis of the number of remaining teeth at the baseline and the depression severity for both follow-ups. In edentulous individuals at baseline, the odds for each of the comparisons “mild vs no”, “moderate vs no”, “moderately severe vs no” and “severe vs no” were above 1, at both follow-ups, but the 95% CI around the ORs across comparisons include the null (not statistically significant). For the categories “1–9” or “10–19” the OR values were less consistent, with some values above 1 and some not. In edentulous individuals, the odds of “moderately severe” or “severe” depression were even higher at both follow-ups. For example, individuals without any teeth at baseline had 3.43 times the odds of “moderately severe” depression at 4 years follow-up (OR 3.43, 95% CI 1.43–8.23).

**Table 3:** Summary of findings of the multinomial logistic regression analysis of the number of remaining teeth at the baseline and the depression severity at follow-up 1 (2 years) and follow-up 2 (4 years)

number of remaining teeth *(baseline)*	*follow-up 1 (2 years) Depression severity*			
	Mild vs. no (OR and CI 95%)	Moderate vs. no (OR and CI 95%)	moderately severe vs. no (OR and CI 95%)	severe vs. no (OR and CI 95%)
none	1.45 (1.01–2.09)	1.11 (0.65–1.93)	1.25 (0.58–2.67)	1.40 (0.52–3.76)
1–9	0.92 (0.69–1.23)	0.94 (0.62–1.43)	1.56 (0.91–2.70)	0.96 (0.41–2.22)
10–19	1.25 (1.01–1.57)	1.51 (1.11–2.05)	1.06 (0.65–1.74)	1.43 (0.73–2.78)
≥ 20	1	1	1	1
	*follow-up 2 (4 years) Depression status*			
none	1.03 (0.66–1.62)	1.91 (1.01–3.66)	3.43 (1.43–8.23)	1.86 (0.46–7.51)
1–9	0.90 (0.65–1.25)	1.45 (0.89–2.37)	1.60 (0.76–3.36)	0.78 (0.22–2.80)
10–19	0.87 (0.67–1.13)	1.05 (0.69–1.57)	1.47 (0.82–2.61)	0.76 (0.27–2.16)
≥ 20	1	1	1	1

The model was adjusted for age, sex, educational attainment, diabetes mellitus II and stressful event at follow-up. OR: odds ratio. CI: confidence interval

## Discussion

In this longitudinal prospective cohort study, individuals with less than 20 teeth in the mouth showed higher odds of incident depression at 2- and 4-years follow-up but is important to notice that most of these values were not statistically significant. Also, edentulism could be associated with increased odds of incident depression at 4-years follow-up and with higher levels of severity of depressive symptoms, which followed an increasing gradient. The findings of this study were consistent with previous studies estimating the association between oral health and depressive symptoms. Yamamoto et al. 2017, in a Japanese longitudinal study, reported that having no teeth and oral health problems may play a role in the development or worsening of depressive symptoms [23]. Zhang et al. (2021), in a longitudinal study with 4 years of follow up, reported that Chinese older adults with fewer teeth left (< 20) and those who were non-denture users were associated with severe depressive symptoms [24]. Also, Kunrath & Ribeiro (2020), reported that Brazilian older adults who experienced tooth loss were more likely to exhibit depressive symptoms [25]. Furthermore, Matsuyama et al. 2021, identified the causal effect of tooth loss on depression among U.S. adults in a natural experiment study, using instrumental analysis (instrumental variable was exposure to drinking water fluoride) in 2006, 2008 or 2010 waves of the Behavioral Risk Factor Surveillance System (BRFSS). They found that for each additional tooth loss, depressive symptoms according to the eight-item Patient Health Questionnaire depression (PHQ-8) score increased by 0.146 (95% CI 0.008–0.284) and the probability of having clinical depression (PHQ-8 higher than 10) increased by 0.81% points (95% CI -0.12 to 1.73). Also, losing ten or more teeth was comparable to adults with major depressive disorder not receiving anti-depressant drugs [17]. Additionally, an Indian cross-sectional study reported that edentulism was associated with poor self-rated health and low psychological and subjective well-being among older adults [26].

Deciphering the potential pathways between oral and mental health outcomes is important to prevent and treat these conditions and for the public health agenda to prioritize mental and oral health. From oral diseases to subsequent depression, it has been hypothesized that neuroinflammation due to past periodontal inflammation or autonomic nerve imbalance due to oral pain, stress and discomfort increases depressive symptoms [27]. On the other hand, although an association between depression and poor oral health habits has been observed, the mechanism of this relationship is not well determined. Also, different mechanisms have been proposed, including changes in behaviors regarding psycho-immunological changes, health management itself, or even a combination of both mechanisms [28]. Depression may affect oral health status, self-perceived oral health, dental attendance, and acquisition and reinforcement of poor oral health behaviors, decreasing the frequency of tooth brushing and flossing [27]. Concerning the biological component, an association between depression and reduction in salivary flow, subjective oral dryness, and downregulation of the immune system has been reported. Consequently, hyposalivation and induced changes in salivary immunity increase the risk of developing oral diseases, especially dental caries or periodontal disease [27].

Our study found heterogeneity in the association between the number of remaining teeth and the incidence of depression when considering men versus women, within and between both follow-ups. The findings in women are more consistent with the hypothesis that fewer number of teeth may increase the risk of depression. Our proposed difference for gender is under evidence that women perceive oral health to be generally more relevant than men [29]. However, our point contrasts with findings of Matsuyama et al. (2021), who observed that the effect of tooth loss on depressive symptoms was larger in men than in women [17]. In addition, other studies have found heterogeneity according to the levels of tooth loss. Zhang et al. (2021), found that the depressive symptom scores of men were higher than those of women concerning to people with nine teeth or less, and the scores of women were higher than those of men for participants with 10–19 teeth [24]. In Chile, according to the last National Health Survey (ENS 2016–2017), the median number of teeth was lower, and the prevalence of depression was higher in women, which also occurred in rural populations like the one included in this cohort [30]. Differences in the trajectories of tooth loss between men and women, which are not fully addressed in this study, may influence the incidence of depression. Finally, regarding the gender gap in depression, there are factors related to susceptibility (biological and psychological) and environmental factors that operate on both the micro and macrolevel [31].

Our findings should be interpreted with caution because there were some limitations. Firstly, the external validity of MAUCO´s findings are limited, since this cohort represents an agricultural population from a small city in the country. Secondly, even we used several confounders including variables at baseline and follow-ups, the risk of residual confounding is likely to affect our conclusions given the complexity of the association under study. For example, in our models, we have considered the variable Diabetes Mellitus II because evidence supports that individuals with diabetes mellitus II have an increased risk of periodontitis or caries, conditions that lead to tooth loss [6]. In addition, diabetes mellitus II, included in metabolic syndrome, has been associated with an increased risk of depression attributable to systemic inflammation [32, 33]. However, there are other inflammatory diseases such as cardiovascular or autoimmune diseases associated with depression [33] that could be affecting our findings and whose complex pathways need to be addressed in future research. Also, the effect of prosthetics or others dental interventions were not captured in our analysis, in fact any potential oral rehabilitation effort received by the participants from the baseline observation to outcome assessment was not determined. Thirdly, there are subjects who were not included in both follow-ups due to lack of information on the depression variable, given the absence of the PHQ-9 register, which may result in selection bias. Fourthly, the number of subjects with “moderately severe” or “severe” depression was around 5% at both follow-ups, which may limit the power of the analyses, despite our sample size was relatively large. Fifthly, the dental examination was performed by dental technicians instead dental professionals which is considered the gold standard. Additionally, by the inherent nature of the outcome, those with more severe depression may have dropped out of the cohort, so the reported findings may be underestimated. Another limitation is that in both follow-ups, a subgroup of the cohort was not re-examined with the PHQ-9 questionnaire, corresponding to around a 5% of the sample in each time, which may introduce a selection bias. The above is related to the fact that our study was nested in a cohort whose objective was the follow-up of multiple noncommunicable diseases (NCDs), focusing on biomedical outcomes and not being primarily a mental health cohort. Finally, we focused on the number of remaining teeth at baseline rather than tooth loss at follow-up to explain the incidence or severity of depression. Future studies may also assess the effect of becoming edentulous during follow-up or the time-varying tooth loss on subsequent changes in the levels of depression. Despite these limitations, the main strength of this study was the longitudinal design, which allow to verify temporal relationship between baseline number of remaining teeth and incident depression over a 2- or 4-years follow-up period. The prospective nature allowed the exclusion of individuals with depression at baseline, which means outcome-free cohort analysis. Future studies should consider other variables, such as the use of antidepressants, from two scientific perspectives. On the one hand, in terms of the association presented in this study, these drugs are designed to decrease depressive symptoms and those, may affect the impact of tooth loss on depression. On the other hand, previous studies have indicated that the use of some antidepressants decreases salivary flow rate, which could increase the risk of caries and thus tooth loss. Both hypotheses have not been measured in our study [27]. Finally, additional investigations may evaluate the role of dental interventions as dental prostheses in the association between the number of the remaining teeth and subsequent depression risk.

## Conclusion

Individuals with less than 20 teeth in the mouth could had higher odds of incident depression at 2- and 4-years follow-up, with differences between men and women. Also, in our study, edentulism was associated with increased odds of incident depression at 4-years follow-up in women, and with higher levels of severity of depressive symptoms. Prevention of tooth loss may potentially reduce the risk or severity of depression in adults.

### Electronic supplementary material

Below is the link to the electronic supplementary material.

Supplementary Material 1

## Data Availability

The data that support the findings of this study are available from the Advanced Center for chronic Diseases (ACCDiS) Directorate in accordance with the local institutional review board authorization, but restrictions apply to the availability of these data, which were used under license for the current study, and so are not publicly available. Data are however available from the authors upon reasonable request and with permission of cferrec@med.puc.cl or by contacting the corresponding author Duniel Ortuño at drortuno@uc.cl. Applications for data usage are accepted online from the MAUCO website: www.mauco.org. Additional information can be requested at contacto@mauco.org or by contacting the corresponding author Duniel Ortuño at drortuno@uc.cl.
