# Supplementary material for: Association between number of remaining teeth and incident depression in a rural Chilean cohort
Source: BMC Oral Health. 2023 Sep 4;23:633. doi: 10.1186/s12903-023-03374-4 (PMC10478404; doi:10.1186/s12903-023-03374-4)
Supplement: Supplementary file 1 — Supplementary Material 1 [file 12903_2023_3374_MOESM1_ESM.docx]

**Supplementary Material**

Supplementary table 1 (S.1): Baseline characteristics of the analytical sample.

|  | **Depression** | | | |  |
| --- | --- | --- | --- | --- | --- |
|  | **Yes** | | **No** | |  |
|  | **N** | **%** | **N** | **%** | **Total** |
| **Number of teeth** |  |  |  |  |  |
| ≥20 teeth | 762 | 14.1 | 4630 | 85.9 | 5392 |
| 10-19 teeth | 368 | 16.4 | 1870 | 83.6 | 2238 |
| 1-9 teeth | 215 | 16.8 | 1061 | 83.2 | 1276 |
| 0 teeth | 99 | 18.3 | 443 | 81.7 | 542 |
| **Sex** |  |  |  |  |  |
| Men | 363 | 8.4 | 3933 | 91.6 | 4296 |
| Women | 1081 | 20.9 | 4071 | 79,1 | 5152 |
| **Age** |  |  |  |  |  |
| 38-45 Years | 366 | 15.2 | 2036 | 84.8 | 2402 |
| 46-53 Years | 416 | 16.3 | 2134 | 83.7 | 2550 |
| 54-61 Years | 366 | 15.8 | 1945 | 84.2 | 2311 |
| 62-69 Years | 214 | 13.3 | 1389 | 86.7 | 1603 |
| ≥70 Years | 82 | 14.1 | 500 | 85.9 | 582 |
| **Education attainment** |  |  |  |  |  |
| ≥12 years of formal Education | 737 | 16.6 | 3693 | 83.4 | 4430 |
| 9-11 years of formal Education | 602 | 14.6 | 3508 | 85.4 | 4110 |
| ≤8 years of formal Education | 91 | 10.6 | 761 | 89.4 | 852 |
| **Diabetes Mellitus II** |  |  |  |  |  |
| Yes | 213 | 19.6 | 869 | 80.4 | 1082 |
| No | 1231 | 14.7 | 7135 | 85.3 | 8366 |

*56 individuals not reported education attainment at baseline.

Supplementary table 2 (S.2): Descriptive statistics of the number of remaining teeth at baseline and depression severity at follow up 1 (2 years) and follow up 2 (4 years) in women, excluding individuals with depression at baseline (PHQ-9>10).

| number of remaining teeth  *(baseline)* | *follow-up 1 (2 years) Depression severity* | | | | | total |
| --- | --- | --- | --- | --- | --- | --- |
|  | no (n/ %) | Mild (n/ %) | Moderate  (n/ %) | moderately severe (n/ %) | severe (n/ %) |  |
| none | 112/ 54.4 | 59/ 28.6 | 19/ 9.2 | 9/ 4.4 | 7/ 3.4 | 206 |
| 1-9 | 261/ 64.3 | 75/ 18.5 | 37/ 9.1 | 26/ 6.4 | 7/ 1.7 | 406 |
| 10-19 | 317/ 54.8 | 152/ 26.3 | 73/ 12.6 | 21/ 3.6 | 16/ 2.7 | 579 |
| ≥20 | 670/ 59.3 | 265/ 23.5 | 121/ 10.7 | 53/ 4.7 | 20/ 1.8 | 1 129 |
| total | 1 360/ 58.6 | 551/ 23.8 | 250/ 10.8 | 109/ 4.7 | 50/ 2.1 | 2 320 |
|  | *follow-up 2 (4 years) Depression severity* | | | | |  |
| none | 91/ 59.5 | 31/ 20.3 | 17/ 11.1 | 10/ 6.5 | 4/ 2.6 | 153 |
| 1-9 | 218/ 65.2 | 67/ 20.1 | 31/ 9.3 | 14/ 4.2 | 4/ 1.2 | 334 |
| 10-19 | 308/ 64.3 | 102/ 21.3 | 39/ 8.1 | 24/ 5.0 | 6/ 1.3 | 479 |
| ≥20 | 537/ 62.3 | 212/ 24.6 | 71/ 8.2 | 29/ 3.4 | 13/ 1.5 | 862 |
| total | 1 154/ 63.1 | 412/ 22.6 | 158/ 8.6 | 77/ 4.2 | 27/ 1.5 | 1 828 |

Supplementary table 3 (S.3): Descriptive statistics of the number of remaining teeth at baseline and depression severity at follow up 1 (2 years) and follow up 2 (4 years) in men, excluding individuals with depression at baseline (PHQ-9>10).

| number of remaining teeth  *(baseline)* | *follow-up 1 (2 years) Depression severity* | | | | | total |
| --- | --- | --- | --- | --- | --- | --- |
|  | no (n/ %) | Mild (n/ %) | Moderate  (n/ %) | moderately severe (n/ %) | severe (n/ %) |  |
| none | 42/ 75.0 | 8/ 14.2 | 3/ 5.4 | 3/ 5.4 | 0/ 0.0 | 56 |
| 1-9 | 114/ 76.0 | 24/ 16.0 | 5/ 3.3 | 4/ 2.7 | 3/ 2.0 | 150 |
| 10-19 | 212/ 74.1 | 43/ 15.0 | 22/ 7.7 | 7/ 2.5 | 2/ 0.7 | 286 |
| ≥20 | 416/ 79.5 | 80/ 15.3 | 20/ 3.8 | 4/ 0.8 | 3/ 0.6 | 523 |
| total | 784/ 77.2 | 155/ 15.3 | 50/ 4.9 | 18/ 1.8 | 8/ 0.8 | 1 015 |
|  | *follow-up 2 (4 years) Depression severity* | | | | |  |
| none | 27/ 75.0 | 7/ 19.4 | 1/ 2.8 | 1/ 2.8 | 0/ 0.0 | 36 |
| 1-9 | 81/ 79.4 | 15/ 14.7 | 6/ 5.9 | 0/ 0.0 | 0/ 0.0 | 102 |
| 10-19 | 161/ 81.3 | 28/ 14.2 | 8/ 4.0 | 1/ 0.5 | 0/ 0.0 | 198 |
| ≥20 | 238/ 80.1 | 41/ 13.8 | 13/ 4.4 | 5/ 1.7 | 0/ 0.0 | 297 |
| total | 507/ 80.1 | 91/ 14.4 | 28/ 4.4 | 7/ 1.1 | 0/ 0.0 | 633 |

Supplementary table 4 (S.4): Summary of findings of the multinomial logistic regression analysis of the number of remaining teeth at the baseline and the depression severity at follow-up 1 (2 years) and follow-up 2 (4 years), in women.

| number of remaining teeth | *follow-up 1 (2 years) Depression severity* | | | |
| --- | --- | --- | --- | --- |
|  | Mild *vs.* no  (OR and CI 95%) | Moderate *vs*. no (OR and CI 95%) | moderately severe *vs.* no  (OR and CI 95%) | severe *vs.* no  (OR and CI 95%) |
| none | 1.49 (1.01-2.23) | 0.96 (0.53-1.74) | 1.01 (0.44-2.32) | 1.75 (0.63-4.88) |
| 1-9 | 0.82 (0.58-1.14) | 0.83 (0.53-1.30) | 1.33 (0.74-2.39) | 0.80 (0.30-2.12) |
| 10-19 | 1.27 (0.99-1,66) | 1.31 (0.93-1.85) | 0.84 (0.48-1.46) | 1.57 (0.77-3.21) |
| ≥20 | 1 | 1 | 1 | 1 |
|  | *follow-up 2 (4 years) Depression severity* | | | |
| none | 0.92 (0.56-1.53) | 2.10 (1.06-4.19) | 4.08 (1.62-10.33) | 1.83 (0.45-7.44) |
| 1-9 | 0.84 (0.58-1.21) | 1.43 (0.84-2.46) | 2.04 (0.95-4.40) | 0.77 (0.22-2.78) |
| 10-19 | 0.83 (0.62-1.13) | 1.07 (0.68-1.67) | 1.80 (0.98-3.31) | 0.77 (0.27-2.17) |
| ≥20 | 1 | 1 | 1 | 1 |

*The model was adjusted for age, sex, educational attainment, diabetes mellitus II and stressful event at follow-up. OR: odds ratio. CI: confidence interval.*

Supplementary table 5 (S.5): Summary of findings of the multinomial logistic regression analysis of the number of remaining teeth at the baseline and the depression severity at follow-up 1 (2 years) and follow-up 2 (4 years), in men.

| number of remaining teeth | *follow-up 1 (2 years) Depression severity* | | | |
| --- | --- | --- | --- | --- |
|  | Mild *vs.* no  (OR and CI 95%) | Moderate *vs*. no (OR and CI 95%) | moderately severe *vs.* no  (OR and CI 95%) | severe *vs.* no  (OR and CI 95%) |
| none | 1.15 (0.48-2.74) | 2.50 (0.60-10.47) | 5.54 (0.76-40.89) | - |
| 1-9 | 1.29 (0.73-2.29) | 1.60 (0.51-4.96) | 4.46 (0.88-22.69) | 1.47 (0.23-9.27) |
| 10-19 | 1.12 (0.72-1.76) | 2.82 (1.37-5.84) | 3.68 (0.95-14.30) | 0.66 (0.10-4.47) |
| ≥20 | 1 | 1 | 1 | 1 |
|  | *follow-up 2 (4 years) Depression severity* | | | |
| none | 1.77 (0.64-4.90) | 0.73 (0.08-6.71) | 1.01 (0.07-13.29) | - |
| 1-9 | 1.20 (0.57-2.56) | 1.44 (0.43-4.78) | - | - |
| 10-19 | 1.05 (0.59-1.88) | 0.87 (0.32-2.36) | 0.16 (0.02-1.54) | - |
| ≥20 | 1 | 1 | 1 | 1 |

*The model was adjusted for age, sex, educational attainment, diabetes mellitus II and stressful event at follow-up. OR: odds ratio. CI: confidence interval.*
